# ALIVIADO DEMENTIA CARE-HOSPICE EDITION: FEASIBILITY AND ACCEPTABILITY RESULTS OF A TWO-PHASED PILOT STUDY

**DOI:** 10.1093/geroni/igz038.484

**Published:** 2019-11-08

**Authors:** Shih-Yin Lin, Alycia A Bristol, Catherine E Schneider, Kimberly Convery, Victor Sotelo, Abraham A Brody

**Affiliations:** 1 NYU Rory Meyers College of Nursing, New York, New York, United States; 2 New York University, New York, New York, United States; 3 NYU Rory Meyers College of Nursing, New York, United States

## Abstract

Limited work has been performed in helping hospice agencies to care for persons with dementia (PWD) and their caregivers in an evidence-based manner despite the increasing number of PWD cared for in this setting. To change the culture of care for PWD and their caregivers receiving hospice, we adapted Aliviado Dementia Care, an evidenced-based interdisciplinary quality improvement program, for use in hospice. The purpose of this pilot study is to examine feasibility and applicability of implementing the Aliviado Dementia Care-Hospice Edition sequentially in 2 hospice agencies in preparation for a nation-wide pragmatic trial. In the first pilot, concluded in March 2019, seven hospice interdisciplinary clinicians were trained as program champions and completed a two-day in-person intensive training on dementia symptom assessment and management, and quality improvement processes. Additionally, 47 interdisciplinary team (IDT) members were provided training via a 5-hour, online program covering dementia symptom assessment and management. All champions trained (100%) reported being satisfied/very satisfied with the program and agreed that the training is applicable to hospice practices. All IDT members who completed the online training (100%) reported being satisfied/very satisfied with the program quality, or agreed/strongly agreed that the content was relevant. The high rates of satisfaction and applicability, reported by the hospice champions and IDT members, provided preliminary evidence supporting the feasibility and applicability of the Aliviado Dementia Care-Hospice Edition.

